# The AMP-Activated Protein Kinase KIN10 Is Involved in the Regulation of Autophagy in *Arabidopsis*

**DOI:** 10.3389/fpls.2017.01201

**Published:** 2017-07-10

**Authors:** Liang Chen, Ze-Zhuo Su, Li Huang, Fan-Nv Xia, Hua Qi, Li-Juan Xie, Shi Xiao, Qin-Fang Chen

**Affiliations:** ^1^State Key Laboratory of Biocontrol and Guangdong Provincial Key Laboratory of Plant Resources, School of Life Sciences, Sun Yat-sen UniversityGuangzhou, China; ^2^College of Life Sciences, South China Agricultural UniversityGuangzhou, China

**Keywords:** ATG1, AMPK, autophagy, KIN10, phosphorylation

## Abstract

Autophagy is a highly conserved system in eukaryotes for the bulk degradation and recycling of intracellular components. Autophagy is involved in many physiological processes including development, senescence, and responses to abiotic and biotic stress. The adenosine 5’-monophosphate (AMP)-activated protein kinase AMPK positively regulates autophagy in mammals; however, the potential function of AMPK in plant autophagy remains largely unknown. Here, we identified KIN10, a plant ortholog of the mammalian AMPK, as a positive regulator of plant autophagy and showed that it acts by affecting the phosphorylation of ATG1 (AUTOPHAGY-RELATED GENE 1) proteins in *Arabidopsis*. Transgenic *Arabidopsis* lines overexpressing *KIN10* (*KIN10-OE*) showed delays in leaf senescence, and increased tolerance to nutrient starvation, these phenotypes required a functional autophagy pathway. Consistent with KIN10 having a potential role in autophagy, the nutrient starvation-induced formation of autophagosomes and cleavage of GFP-ATG8e were accelerated in the *KIN10-OE* lines compared to the wild type. Moreover, the *KIN10-OE* lines were less sensitive to drought and hypoxia treatments, compared with wild type. Carbon starvation enhanced the level of phosphorylated YFP-ATG1a in the *KIN10-OE* lines compared to that of wild type. Together, these findings suggest that KIN10 is involved in positive regulation of autophagy, possibly by affecting the phosphorylation of ATG1s in *Arabidopsis*.

## Introduction

Autophagy is the process of degradation and recycling of cytoplasmic organelles, proteins, and macromolecules, and is highly conserved among eukaryotes. Autophagy is activated by a variety of stress factors, such as nutrient deprivation, hypoxia, reactive oxygen species, and infection by pathogen (Kroemer et al., 2010; Han et al., 2011). Autophagy plays an essential role in the maintenance of cellular homeostasis under changing nutrient conditions. Among the three types of autophagy, macroautophagy (hereafter referred to as autophagy) is the predominant form (Klionsky, 2007). During autophagy, double-membrane vesicles, called autophagosomes, are formed from the expanding membranes of preautophagosomal structures; these autophagosomes sequester the enclosed components and deliver them to the lysosome/vacuole for degradation.

The serine/threonine protein kinase ULK1(Unc-51-like kinases 1, mammalian homologs of ATG1) activates autophagy in response to developmental cues or stress signals by initiating autophagosome formation (Mizushima, 2010; Wirth et al., 2013; Wong et al., 2013). In mammalian cells, ULK1 activity is directly controlled by the target of rapamycin (TOR) and the AMP-dependent protein kinase (AMPK) (Kim et al., 2011; Shang and Wang, 2011; Alers et al., 2012). Under nutrient-rich conditions, the activated TOR kinase disrupts the ULK1-ATG13 complex by phosphorylating the ATG13 subunit, and thereby inhibits autophagy. However, under nutrient starvation conditions, AMPK directly phosphorylates ULK1 at the Ser 317 and Ser 777 residues, subsequently activating autophagy (Kim et al., 2011). Alternatively, AMPK may activate autophagy by inhibiting TORC1 (TOR complex 1) activity (Gwinn et al., 2008; Lee et al., 2010). ULK1 may also be involved in the termination of autophagy. Specifically, ULK1 represses AMPK activity through a negative regulatory feedback loop (Löffler et al., 2011). Similarly, another study suggests that the KLHL20-mediated ubiquitination and degradation of ULK1 contributes to the termination of autophagy (Liu et al., 2016).

In plants, the Snf1-related kinase 1 (SnRK1), a homolog of the yeast Snf1 and mammalian AMPK, is a highly conserved energy sensor and is activated under energy deprivation (Polge and Thomas, 2007; Baena-González and Sheen, 2008; Emanuelle et al., 2015). SnRK1 is composed of one catalytic α subunit (KIN10 and 11 in *Arabidopsis*) and two regulatory subunits, β and γ (Polge and Thomas, 2007; Emanuelle et al., 2015). Overexpression of *KIN10* delays flowering and leaf senescence in *Arabidopsis*, suggesting that KIN10 play a positive role in the regulation of growth and development as well as energy signaling (Baena-González et al., 2007). Although Snf1/AMPK likely promotes autophagy by directly activating ATG1/ULK1 in yeast and animals, the role of SnRK1 in plant autophagy is not well characterized.

Our study demonstrated that *Arabidopsis* KIN10 is a positive regulator of autophagy. Under nutrient starvation, transgenic plants overexpressing *KIN10* (*KIN10-OE*) showed enhanced autophagosome formation and increased tolerance to nutrient deprivations. Furthermore, the level of starvation-induced phosphorylation of ATG1 increased in the *KIN10-OE* lines, suggesting that KIN10 is likely involved in positive regulation of autophagy, possibly by affecting the phosphorylation of ATG1 proteins.

## Materials and Methods

### Plant Materials, Growth Conditions, and Treatments

The *KIN10* overexpression lines (*OE-1* and *OE-2*) and *KIN10 RNAi* lines (*RNAi-1* and *RNAi-7*) used in this study were in the *Arabidopsis* Landsberg *erecta* (L*er*) background (Baena-González et al., 2007). The autophagy-related mutants *atg5-1* and *atg7-3* (Thompson et al., 2005; Lai et al., 2011; Chen et al., 2015; Col-0 ecotype) were backcrossed twice to the L*er* wild-type plants to obtain *atg5-L* and *atg7-L* plants. The *atg5-L* and *atg7-L* mutants were further crossed to the *OE-1* line to generate *OE-1 atg5-L* and *OE-1 atg7-L* lines. Transgenic lines expressing *GFP-ATG8e* and *YFP-ATG1a*, driven by the CaMV 35S promoter, have been previously described (Xiao et al., 2010; Suttangkakul et al., 2011). All *Arabidopsis* seeds were surface-sterilized with 20% bleach containing 0.1% Tween-20 for 20 min, and then washed 5 times with sterile water. Seeds were sown on Murashige and Skoog (MS) medium, followed by cold treatment in the dark for 3 days. Seven days after germination, seedlings were transplanted into soil and grown in a plant growth room with a16-h-light/8-h-dark cycle at 22°C.

For the carbon starvation treatment, 7-day-old MS-grown seedlings or 4-week-old soil-grown plants were transferred to continuous darkness for the indicated duration followed by recovery under normal growth conditions for 7 days. Samples were collected or photographed at the indicated time points. To calculate the survival rate after darkness, at least 18 plants per genotype were dark-treated followed by a 7-day recovery. The number of surviving plants, where survival if defined as the capability to produce new leaves, was recorded. For the nitrogen starvation treatment, 7-day-old seedlings grown on MS medium were transferred to solid MS or nitrogen-deficient solid MS medium and grown for 5 days. Chlorophyll contents were measured and calculated after the recovery.

### RNA Extraction and Quantitative Reverse-Transcription PCR Analysis

Total RNA extraction and quantitative reverse-transcription PCR (qRT-PCR) analysis were performed as previously described (Chen et al., 2015). Briefly, the isolated RNA was reverse transcribed using the PrimeScript RT Reagent Kit with gDNA Eraser (Takara, RR047A) following the manufacturer’s instructions. The qPCR was carried out using SYBR Green master mix (Takara, RR420A) on a StepOne Plus real-time PCR system (Applied Biosystems). The conditions for the qPCR were: initial denaturation at 95°C for 5 min followed by 40 cycles of PCR (denaturing at 95°C for 10 s, annealing at 55°C for 15 s, and extension at 72°C for 30 s). Three experimental replicates were used for each reaction. *ACTIN2* was used as the reference gene. Gene-specific primers used for the qPCR analysis are listed in Supplementary Table S1.

### Laser Scanning Confocal Microscopy

The stable transgenic lines expressing a GFP-ATG8e fusion protein were used to monitor autophagosome formation (Xiao et al., 2010). Seven-day-old *GFP-ATG8e* seedlings grown in MS solid medium were transferred to MS medium or MS medium lacking sugars (MS-C) under darkness for the indicated times. After treatment, the primary root cells were observed using an LSM 780 NLO laser scanning confocal microscope (Carl Zeiss).

### Western Blot Analysis

Total protein extraction was performed as previously described (Chen et al., 2015). Briefly, 4-week-old plant leaves or 7-day-old whole seedling were ground in liquid nitrogen and homogenized in ice-cold extraction buffer (50 mM sodium phosphate, pH 7.0, 200 mM NaCl, 10 mM MgCl_2_, 0.2% β-mercaptoethanol and 10% glycerol) supplemented with protease inhibitor cocktail (Roche, 04693132001). Total homogenates were placed on ice for 30 min, and then centrifuged for 30 min at 11,000 *g*. The supernatant was transferred to a new microfuge tube for SDS-PAGE electrophoresis.

For immunoblot analysis, total proteins were subjected to SDS-PAGE and electrophoretically transferred to a Hybond-C membrane (Amersham, 10600016). Anti-GFP antibodies were used to detect GFP as previously described (Chen et al., 2015). YFP was detected with rabbit anti-GFP polyclonal antibodies (Abcam, ab290).

### Phosphatase Treatment

Phosphatase treatment was performed according to Suttangkakul et al. (2011) with minor modification. Seven-day-old *YFP-ATG1a* and *YFP-ATG1a*/*KIN10-OE* seedling were homogenized in ice-cold protein extraction buffer supplemented with 1 mM phenylmethylsulfonyl fluoride and protease inhibitor cocktail (Roche). Samples were placed on ice for 30 min, and then centrifuged for 30 min at 11,000 *g*. The supernatant was incubated with λ protein phosphatase (New England Biolabs) with or without addition of phosphatase inhibitor PhosSTOP (Roche) for 30 min at 30°C. 2 × SDS-PAGE sample buffer was added to the total sample and heated to 95°C for 5 min.

### Statistical Analysis

Data are reported as means ± SD of three independent experiments unless otherwise indicated. The significance of the differences between groups was determined by a two-tailed Student’s *t*-test. *P*-values < 0.05 or < 0.01 were considered significant.

### Accession Numbers

Sequence data from this article can be found in the Arabidopsis Genome Initiative or GenBank databases under the following accession numbers: *KIN10* (At3g01090), *ATG1a* (At3g61960), *ATG1b* (At3g53930), *ATG1c* (At2g37840), *ATG2* (At3g19190), *ATG5* (At5g17290), *ATG6* (At3g61710), *ATG7* (At5g45900), *ATG8a* (At4g21980), *ATG8e* (At2g45170), *ATG9* (At2g31260), *ATG10* (At3g07525), *ATG13a* (At3g49590), *ATG13b* (At3g18770), *ATG18a* (At3g62770), and *PI3K* (At1g60490).

## Results

### Transgenic Plants Overexpressing *KIN10* Showed Delayed Leaf Senescence and Enhanced Tolerance to Nutrient Starvations

**FIGURE 1:**
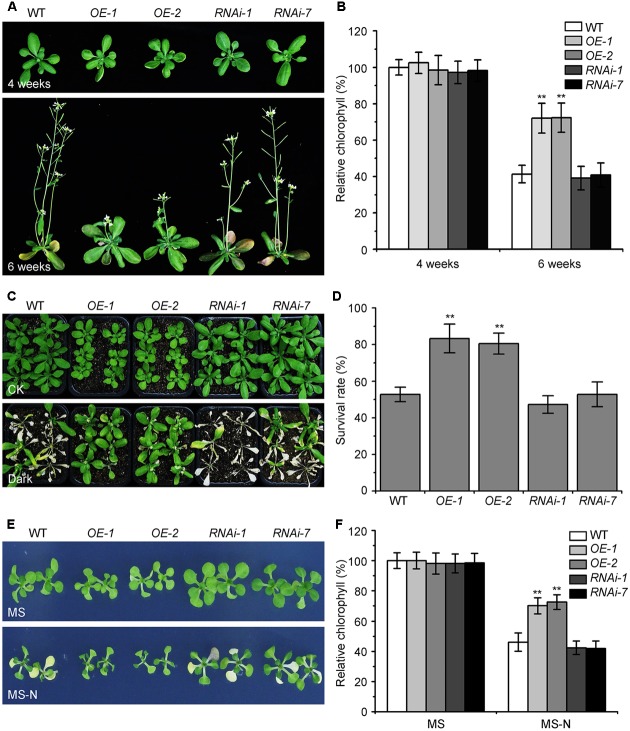
Overexpression of *KIN10* delays senescence and enhances tolerance to nutrient starvation in *Arabidopsis*. **(A)** Naturally induced senescence of the wild type (WT) and the *KIN10-OE* (*OE-1* and *OE-2*) and *KIN10-RNAi* lines (*RNAi-1* and *RNAi-7*). Photos were taken at 4 and 6 weeks after germination. **(B)** Relative chlorophyll contents in the leaves of 4- and 6-week-old WT, *OE-1*, *OE-2*, *RNAi-1*, and *RNAi-7* plants. The data are means ± SD (*n* = 3) calculated from three biological replicates. For each experiment, a population of 14 plants was recorded per genotype. ^∗∗^*P* < 0.01 by Student’s *t*-test. **(C)** Phenotypes of the WT, *OE-1*, *OE-2*, *RNAi-1*, and *RNAi-7* plants after carbon starvation. Four-week-old WT, *OE-1*, *OE-2*, *RNAi-1*, and *RNAi-7* plants grown under normal growth conditions were transferred to constant darkness for 7 days and photos were taken after a 7-day recovery. **(D)** Survival rates of the WT, *OE-1*, *OE-2*, *RNAi-1*, and *RNAi-7* plants described in **(C)**. The data are means ± SD (*n* = 3) calculated from three biological replicates. For each biological replicate, a population of 18 plants was recorded per genotype. ^∗∗^*P* < 0.01 by Student’s *t*-test. **(E)** Phenotypes of the WT, *OE-1*, *OE-2*, *RNAi-1*, and *RNAi-7* plants after nitrogen starvation. One-week-old WT, *OE-1*, *OE-2*, *RNAi-1*, *RNAi-7* seedlings grown on MS solid medium were transferred to MS or MS-N solid medium and photos were taken after 5 days of treatment. **(F)** Relative chlorophyll contents of the WT, *OE-1*, *OE-2*, *RNAi-1*, and *RNAi-7* seedlings described in **(E)**. The data are means ± SD (*n* = 3) calculated from three biological replicates. For each biological replicate, a population of 20 plants was recorded per genotype. ^∗∗^*P* < 0.01 by Student’s *t*-test.

The KIN10 overexpression lines (*OE-1* and *OE-2*) showed delayed leaf senescence (Baena-González et al., 2007). To examine the potential role of *Arabidopsis* KIN10 in autophagy, we further examined the response of the *KIN10-OE* lines and KIN10 RNA interference lines (*RNAi-1* and *RNAi-7*) to naturally induced senescence and nutrient deficiency. The RNA and protein level of *KIN10* in the *KIN10-OE* and *KIN10-RNAi* lines were first confirmed by qRT-PCR and western blot analyses (**Supplementary Figure S1**). Under normal growth conditions, both *KIN10-OE* lines displayed slower growth and delayed natural senescence compared to the wild type, while the *KIN10-RNAi* lines showed similar phenotypes to the wild-type plants (**Figure 1A**). The level of chlorophyll in the leaves of 6-week-old *KIN10-OE* lines was much higher than that of the wild type (**Figure 1B**).

The *KIN10-OE* lines showed enhanced tolerance to carbon starvation induced by constant darkness for 7 days followed by a 7-day recovery, while the *KIN10-RNAi* lines appeared similar to the wild-type plants after this treatment (**Figures 1C,D**). For the nitrogen deficiency treatment, 7-day-old MS-grown seedlings were transferred to MS or MS-N solid medium for 5 days. The cotyledons of the wild-type plants and *KIN10-RNAi* lines were significantly yellowed as indicated by the reduced chlorophyll contents (**Figures 1E,F**). In contrast, the *KIN10-OE* lines were more resistant to nitrogen deficiency and had significantly higher chlorophyll levels compared to that of the wild type (**Figures 1E,F**). These findings suggest that overexpression of *KIN10* can delay natural senescence and improves tolerance to carbon and nitrogen starvation.

### The Enhanced Tolerance of the *KIN10-OE* Lines to Nutrient Starvation Is Dependent on a Functional Autophagy Pathway

**FIGURE 2:**
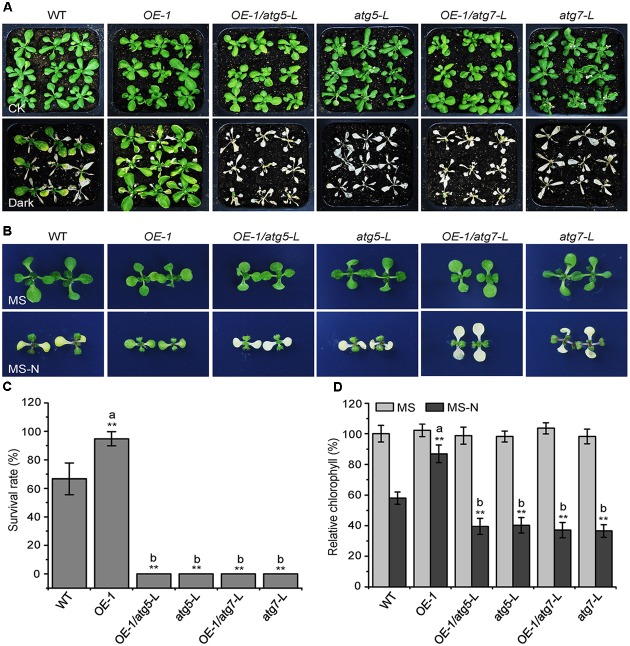
The enhanced tolerance of the *KIN10-OE* lines to nutrient starvation is dependent on a functional autophagy pathway. **(A)** Phenotypes of the wild type (WT), *OE-1*, *OE-1/atg5-L*, *atg5-L*, *OE-1/atg7-L*, and *atg7-L* plants after carbon starvation. Four-week-old WT, *OE1*, *OE-1/atg5-L, atg5-L, OE-1/atg7-L*, and *atg7-L* plants grown under normal conditions were transferred to constant darkness for 7 days and photographs were taken after 7 days of dark treatment. **(B)** Phenotypes of the WT, *OE-1*, *OE-1/atg5-L*, *atg5-L*, *OE-1/atg7-L*, and *atg7-L* plants after nitrogen starvation. One-week-old WT, *OE-1*, *OE-1/atg5-L*, *atg5-L*, *OE-1/atg7-L*, and *atg7-L* seedlings grown on solid MS medium were transferred to MS or MS-N solid medium and photos were taken after 5 days of treatment. **(C)** Survival rate of the WT, *OE-1*, *OE-1/atg5-L*, *atg5-L*, *OE-1/atg7-L*, and *atg7-L* plants described in **(A)**. The data are means ± SD (*n* = 3) calculated from three biological replicates. For each biological replicate, a population of 18 plants was recorded per genotype. ^∗∗^*P* < 0.01 by Student’s *t*-test. **(D)** Relative chlorophyll levels in the leaves of the WT, *OE-1*, *OE-1/atg5-L*, *atg5-L*, *OE-1/atg7-L*, and *atg7-L* seedlings described in **(B)**. The data are means ± SD (*n* = 3) calculated from three biological replicates. For each biological replicate, five technical replicates (each replicate was pooled with 20 seedlings) were measured per genotype. ^∗∗^*P* < 0.01 by Student’s *t*-test. “a” indicates values that are significantly higher than that of the WT; “b” indicates values that are significantly lower than that of the WT.

Given that the *KIN10*-overexpression lines showed delayed leaf senescence and enhanced tolerance to nutrient starvation (**Figures 1**, **2**), and that the autophagy-deficient mutants had the opposite phenotype (Baena-González et al., 2007; Li and Vierstra, 2012), we used these plants to further assess the genetic connection between KIN10 and the autophagy pathway. The *atg5-L* and *atg7-L* mutants (*atg5* and *atg7* mutants in the L*er* background) were crossed to *KIN10-OE-1* (*OE-1*) to generate *OE-1 atg5-L* and *OE-1 atg7-L* lines. We then tested the tolerance of the 4-week-old wild-type, *OE-1*, *OE-1 atg5-L*, *OE-1 atg7-L*, *atg5-L*, and *atg7-L* plants to carbon starvation. Compared to the wild-type plants, the *OE-1* plants showed enhanced tolerance and the *atg5-L* and *atg7-L* plants showed decreased tolerance to carbon starvation. Interestingly, the *OE-1 atg5-L* and *OE-1 atg7-L* lines displayed similar sensitivities to carbon starvation to that of the *atg5-L* and *atg7-L* mutants (**Figure 2A**). In addition, the enhanced resistance of the *OE-1* line to nitrogen deficiency was attenuated by the loss-of-function of *ATG5* and *ATG7* (**Figure 2B**). The enhanced tolerance to starvation in the *OE-1* line was further supported by the higher survival rates (**Figure 2C**) and higher relative chlorophyll contents (**Figure 2D**) in this line. Together, these results indicate that the enhanced tolerance to nutrient starvation in the *KIN10-OE* lines is dependent on a functional autophagy pathway. The evidence that the autophagy-associated phenotypes in the *KIN10-OE*s were primarily recovered by the autophagy deficient mutants, suggesting that autophagy acts downstream of KIN10 to affect plant growth and stress responses. Given that KIN10 is a well-known master regulator in energy signaling in *Arabidopsis*, we therefore proposed that it governs a cellular switch between plant growth and stress responses by modulating various downstream signaling pathways, including autophagy.

### The *KIN10-OE* Lines Are Tolerant to Drought and Submergence

**FIGURE 3:**
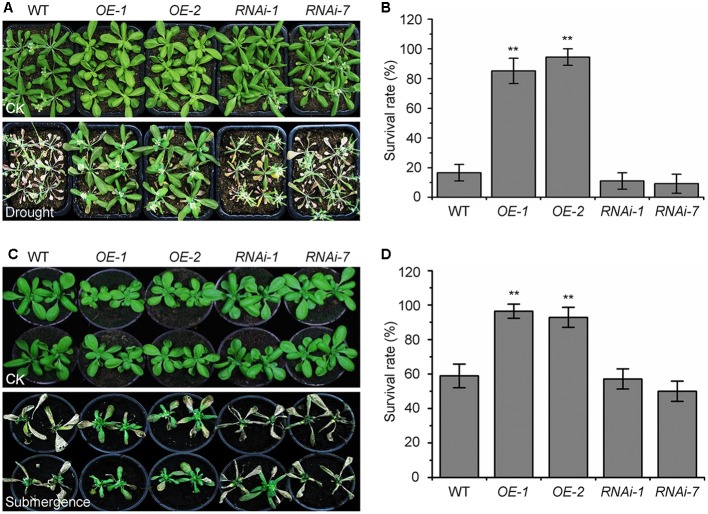
Overexpression of *KIN10* enhances tolerance to drought and submergence. **(A)** Phenotypes of the wild type (WT), *OE-1*, *OE-2*, *RNAi-1*, and *RNAi-7* plants after drought stress. WT, *OE-1*, *OE-2*, *RNAi-1*, and *RNAi-7* plants were grown under normal growth conditions for 3 weeks and then water was withheld for a 14-day drought treatment. Photos were taken after a 4-day recovery. **(B)** Survival rates of the WT, *OE-1*, *OE-2*, *RNAi-1*, and *RNAi-7* plants described in **(A)** after re-watering for 4 days. The data are means ± SD (*n* = 3) calculated from three biological replicates. For each biological replicate, a population of 18 plants was recorded per genotype. ^∗∗^*P* < 0.01 by Student’s *t*-test. **(C)** Phenotypes of the WT, *OE-1*, *OE-2*, *RNAi-1*, and *RNAi-7* plants after the submergence treatment. Four-week-old WT, *OE-1*, *OE-2*, *RNAi-1*, and *RNAi-7* plants were submerged for 6 days and photos were taken after a 6-day recovery. **(D)** Survival rates of the WT, *OE-1*, *OE-2*, *RNAi-1*, and *RNAi-7* plants described in **(C)** after the 6-day recovery. The data are means ± SD (*n* = 3) calculated from three biological replicates. For each biological replicate, a population of 18 plants was recorded per genotype. ^∗∗^*P* < 0.01 by Student’s *t*-test.

The autophagy-defective mutants are hypersensitive to abiotic stresses such as drought and submergence (Liu et al., 2009; Chen et al., 2015). To further assess the role of KIN10 in autophagy-related stress responses, the wild-type (L*er*), the *KIN10-OE* lines (*OE-1* and *OE-2*), and the *KIN10-RNAi* lines (*RNAi-1* and *RNAi-7*) were subjected to drought and submergence treatments. As shown in **Figure 3**, no significant differences were observed between the wild type and the *KIN10-OE* or *KIN10-RNAi* lines under normal growth conditions. However, after a 14-day drought treatment, the leaves of the wild type and the *KIN10-RNAi* lines turned yellow and wilted, while the leaves of the *KIN10-OE* lines remained green and turgid (**Figure 3A**). After a 4-day recovery by rehydration, the survival rates of the *KIN10-OE* lines were significantly higher than those of the wild type and the *KIN10-RNAi* lines (**Figure 3B**). In addition, the *KIN10-OE* lines were much more tolerant than the wild type and the *KIN10-RNAi* plants to a 6-day submergence in water (under light conditions) followed by a 6-day recovery (**Figure 3C**), which was supported by the improved survival rates of the *KIN10-OE* lines compared to the wild-type plants after submergence (**Figure 3D**).

### Overexpression of *KIN10* Activates the Formation of Autophagosomes

To examine the potential involvement of KIN10 in regulating autophagosome formation, we first tested the abundance of *ATG* transcripts (*ATG2*, *ATG5*, *ATG7*, *ATG8a*, *ATG10*, and *ATG18a*) in the wild type, *KIN10-OE* lines (*OE-1* and *OE-2*), and the *KIN10-RNAi* lines (*RNAi-1* and *RNAi-7*). qRT-PCR analyses showed no significant changes in the expression levels of *ATG7*, *ATG10*, and *ATG18a* among the wild type, *KIN10-OE* lines, or the *KIN10-RNAi* lines, while the expression of *ATG2*, *ATG5*, and *ATG8a* was slightly upregulated in the *KIN10-OE* lines in comparison to the wild type (**Supplementary Figure S2**).

**FIGURE 4:**
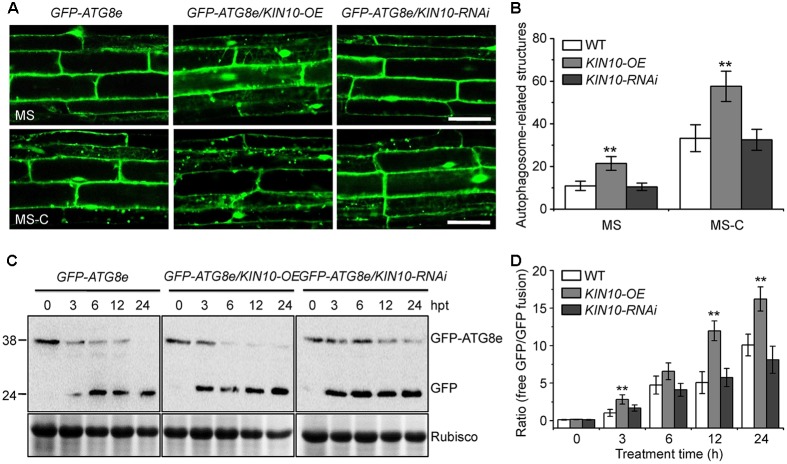
Overexpression of *KIN10* enhances the formation of autophagosomes. **(A)** Microscopic analyses of autophagosome-related structures in the *GFP-ATG8e*, *GFP-ATG8e/KIN10-OE*, and *GFP-8e/KIN10-RNAi* lines. Seven-day-old *GFP-ATG8*, *GFP-ATG8e/KIN10-OE*, and *GFP-8e/KIN10-RNAi* seedlings were grown on MS medium or MS-C medium for 6 h and then visualized by fluorescence confocal microscopy. The punctate structures labeled by green fluorescence from the cleavage of GFP-ATG8e indicate the autophagosome-related structures. Bar = 50 μm. **(B)** Quantification of autophagosome-related structures. Numbers of autophagosome-related structures described in **(A)** were counted using ImageJ software. The data are means ± SD (*n* = 30) calculated from three independent trials. For each trial, 10 independent seedlings were observed per genotype. ^∗∗^*P* < 0.01 by Student’s *t*-test. **(C)** Immunoblot analysis showing the processing of GFP-ATG8e fusion proteins in the *GFP-ATG8e*, *GFP-ATG8e/KIN10-OE*, and *GFP-8e/KIN10-RNAi* lines after the carbon starvation treatment. Seven-day-old *GFP-ATG8e, GFP-ATG8e/KIN10-OE*, and *GFP-8e/KIN10-RNAi* seedlings were grown on MS-C medium for 0, 3, 6, 12, and 24 h. Crude protein extracts were subjected to SDS-PAGE and immunoblot analysis with anti-GFP antibodies. GFP-ATG8e and free GFP bands are indicated on the right. Coomassie blue-stained total proteins (Rubisco) are shown below the blots to indicate the amount of protein loaded per lane. **(D)** Quantification of the free GFP/GFP-ATG8e ratio during carbon starvation by densitometric scans of the immunoblots shown in **(C)**. The data are means ± SD (*n* = 3) calculated from three biological replicates. ^∗∗^*P* < 0.01 by Student’s *t*-test.

To further investigate the role of KIN10 in the induction of autophagy, we examined autophagosome formation in the wild type, *KIN10-OE* and *KIN10-RNAi* lines using green fluorescent protein (GFP)-tagged ATG8e (Xiao et al., 2010). Seven-day-old *GFP-ATG8e* (wild-type background), *GFP-ATG8e/KIN10-OE* and *GFP-ATG8e/KIN10-RNAi* seedlings were transferred to solid MS medium (MS) or MS-C under darkness for 6 h, and the GFP fluorescence of root cells was subsequently observed using confocal microscopy. As shown in **Figure 4**, under both MS and MS-C conditions, the numbers of GFP-ATG8e labeled punctate structures significantly increased in the *KIN10-OE* lines in comparison to the wild type and the *KIN10-RNAi* lines (**Figures 4A,B**). Upon nutrient starvation, the GFP-ATG8e fusion protein is degraded to release a free, relatively stable GFP, and the accumulation of GFP signals reflects the level of induction of autophagy (Li et al., 2014; Klionsky et al., 2016). As shown in **Figure 4C**, the degradation of the GFP-ATG8e fusion protein in the *GFP-ATG8e/KIN10-OE* line was faster than in the *GFP-ATG8e* or *GFP-ATG8e/KIN10-RNAi* line. Consistent with this, the ratio of free GFP to GFP-ATG8e in the *GFP-ATG8e/KIN10-OE* line was higher than that of in the *GFP-ATG8e* or *GFP-ATG8e/KIN10-RNAi* line (**Figure 4D**), suggesting that overexpression of *KIN10* enhances autophagic activity.

### Overexpression of *KIN10* Enhances the Phosphorylation of ATG1 Proteins

**FIGURE 5:**
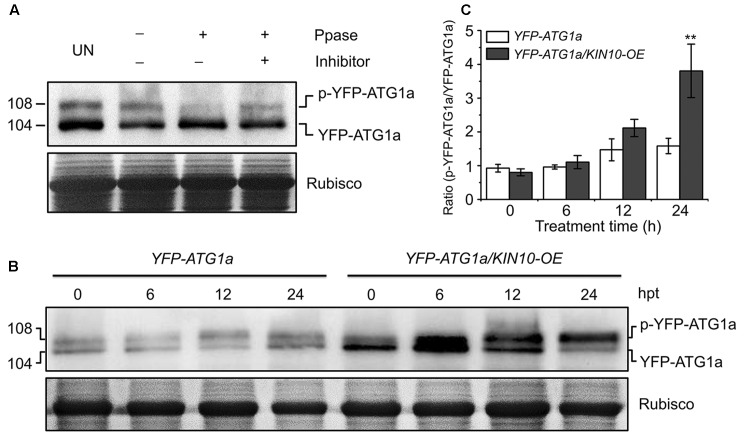
Overexpression of *KIN10* increased the level of ATG1 phosphorylation. **(A)** Effect of λ protein phosphatase on the SDS-PAGE mobility of YFP-ATG1a. Proteins were extracted form 7-day-old *YFP-ATG1a* transgenic plants grown on MS medium. Extracts were treated for 1 h with λ phosphatase (Ppase) with or without the phosphatase inhibitor PhosSTOP and then subjected to immunoblot analysis with anti-GFP antibodies. UN, untreated extracts. **(B)** Immunoblot analysis of ATG1 phosphorylation in the *YFP-ATG1a* and *YFP-ATG1a/KIN10-OE* transgenic plants. Seven-day-old *YFP-ATG1a* and *YFP-ATG1a/KIN10-OE* transgenic plants were grown on MS-C medium for the indicated times prior to protein extraction. Crude extracts were subjected to SDS-PAGE and immunoblot analysis with anti-GFP antibodies. The p-YFP-ATG1a and YFP-ATG1a bands are indicated on the right. Coomassie blue-stained total proteins (Rubisco) are shown below the blots to indicate the amount of protein loaded per lane. **(C)** Quantification of the p-YFP-ATG1a/YFP-ATG1a ratio during carbon starvation by densitometric scans of the immunoblots shown in **(B)**. The data are means ± SD (*n* = 3) calculated from three biological replicates. ^∗∗^*P* < 0.01 by Student’s *t*-test.

Given that AMPK phosphorylates ULK1 to activate autophagy in mammalian cells (Egan et al., 2011; Kim et al., 2011), we hypothesized that KIN10 may be involved in autophagy by directly or indirectly phosphorylating ATG1. To confirm this, ATG1 phosphorylation was first tested using λ protein phosphatase and phosphatase inhibitor PhosSTOP in a yellow fluorescent protein (*YFP*)-tagged *ATG1a* transgenic plant (*YFP-ATG1a*) (Suttangkakul et al., 2011). As shown in **Figure 5A**, two species of YFP-ATG1a were detected using anti-GFP antibodies by western blot. Consistent with a previous study (Suttangkakul et al., 2011), the λ phosphatase treatment of total protein extracted from the *YFP-ATG1a* transgenic plant reduced the levels of the higher molecular weight species, while PhosSTOP blocked this shift (**Figure 5A**), To demonstrate the role of KIN10 in the regulation of ATG1, we crossed the *YFP-ATG1a* line to the *OE-1* line to generate the *YFP-ATG1a*/*KIN10-OE* lines. Immunoblot analysis showed that the level of YFP-ATG1a fusion protein was significantly higher in the *YFP-ATG1a KIN10-OE* line than in the *YFP-ATG1a* line (**Figure 5B**). Upon carbon starvation, the phosphorylation status of ATG1 was enhanced in the *YFP-ATG1a KIN10-OE* line in comparison to the *YFP-ATG1a* line (**Figures 5B,C**).

To determine whether the accumulation of YFP-ATG1a was caused by the higher transcription of *YFP-ATG1a* in the *YFP-ATG1a KIN10-OE* line, we analyzed the total transcript level of *ATG1a* and *YFP-ATG1a* during carbon starvation by qRT-PCR. As shown in **Supplementary Figure S3**, The total transcript level of *ATG1a* was enhanced in the *YFP-ATG1a KIN10-OE* line but not in the *YFP-ATG1a* line, while the *YFP-ATG1a* transcript level did not change much in either line. Interestingly, the total expression of both *ATG1a* and *YFP-ATG1a* was slightly higher in the *YFP-ATG1a KIN10-OE* line than in the *YFP-ATG1a* line.

## Discussion

As a mammalian ortholog of yeast ATG1, ULK1 is phosphorylated by AMPK to activate autophagy or phosphorylated by TOR to repress autophagy (Kim et al., 2011; Shang and Wang, 2011). It has been proposed that AMPK can activate autophagy by directly phosphorylating ULK1 or by suppressing the activity of mTORC1, which thereby inhibits ULK1 activity by phosphorylation (Kim et al., 2011). In this study, we present several lines of evidence to support the hypothesis that KIN10 is involved in the regulation of autophagy in *Arabidopsis*. First, the overexpression of *KIN10* (*KIN10-OE*) resulted in delayed leaf senescence and enhanced tolerance to nutrient deficiencies and abiotic stresses in *Arabidopsis* (**Figures 1**, **3**). Second, the enhanced tolerance to nutrient starvation in the *KIN10-OE* lines is dependent on a functional autophagy pathway (**Figure 2**). Third, the expression of *ATGs* and autophagosome formation and degradation were enhanced in the *KIN10-OE* lines (**Figure 4**). Last, the phosphorylation of ATG1a was enhanced in the *KIN10-OE* lines in comparison to that of the wild type (**Figure 5**). Taken together, these results demonstrate that KIN10 is a positive regulator of autophagy activation, possibly by enhancing the phosphorylation of ATG1, a mechanism that seems to be conserved in plants and animals.

KIN10 is an energy sensor in plants that has diverse functions in the regulation of plant metabolism, development, and stress responses (Polge and Thomas, 2007; Baena-González and Sheen, 2008; Jossier et al., 2009). KIN10 may be essential for maintaining the cell’s energy balance during nutrient starvations (Baena-González et al., 2007). For example, the overexpression of *KIN10* delays natural and nitrogen starvation-induced senescence, and the plants where *KIN10* and *KIN11* have been targeted by virus-induced gene silencing (*KIN11* is a functionally redundant homolog of *KIN10* in *Arabidopsis*) have an early senescence phenotype (Baena-González et al., 2007). Here, we investigated the autophagy-associated senescence phenotypes of the *KIN10-OE* and *KIN10-RNAi* lines and suggested that the phenotypes observed in the *KIN10-OE* lines were genetically linked to the autophagy pathway. Though we observed significant phenotypic differences in the *KIN10-OE* lines compared to the wild type in response to the treatments, we did not observe significant differences in the autophagy-associated phenotypes, gene expression and autophagosome formation in the *KIN10-RNAi* lines after the treatments. It is not feasible to use the *kin10 kin11* virus-induced gene silencing lines in autophagy-related phenotypic analyses due to the severe growth inhibition of these silenced lines (Baena-González et al., 2007). It is still unknown whether KIN11 plays a redundant role in the regulation of autophagy, and generation of transgenic lines with double knockdown of *KIN10* and *KIN11* expression will be useful for future investigation of the functions of KIN10 and KIN11 in autophagy induction.

Autophagy plays an important role in the plant’s response to various stress conditions, such as oxidative stress (Xiong et al., 2007a,b), nutrient deficiency (Doelling et al., 2002), hypoxia (Chen et al., 2015), and pathogen infection (Liu et al., 2005; Wang et al., 2011). The autophagy-defective (*atg*) mutants, such as *atg2-1*, *atg5-1*, *atg7-1*, and *atg10-1*, are frequently used to study the role of autophagy in stress responses in plants. In contrast to the situation in animal systems, little is known about the effects of constitutive activation of autophagy in plants. TOR has been suggested to be a negative regulator of plant autophagy (Liu and Bassham, 2010). To circumvent the embryo lethal phenotype of *TOR* loss-of-function mutant, RNA interference (RNAi) was used to reduce *TOR* transcript levels in the *RNAi-AtTOR* plants, which show constitutive autophagy (Liu and Bassham, 2010). Given the fundamental roles of TOR in plant growth and metabolism (Xiong and Sheen, 2014), it is difficult to distinguish whether the phenotype of the *RNAi-AtTOR* line is caused by increased autophagy or is due to the reduced expression of *TOR*. In comparison, we suggested that overexpression of *KIN10* enhances tolerance to nutrient deficiencies in *Arabidopsis* (**Figure 1**), and this enhanced tolerance was blocked by a deficiency in autophagy (**Figure 2**), which demonstrates that KIN10 improves tolerance to nutrient starvations by directly activating the autophagy pathway. Moreover, we showed that the *KIN10-OE* lines were more tolerant to drought and submergence (**Figure 3**), indicating that *KIN10* is a potential candidate for genetic improvement of plant responses to nutrient deficiencies and water-related stresses. However, we cannot exclude the possibility that KIN10 may indirectly regulate autophagy by inhibiting TOR, since the mammalian AMPK has been reported to regulate autophagy by negative modulation of mTORC1 (Kimura et al., 2003). Further investigations of the coordinated functions of KIN10 and TOR will deepen our understanding of the upstream energy signals that regulate autophagy initiation in *Arabidopsis*.

The roles of AMPK in the regulation of autophagy have been extensively studied in animals (Gwinn et al., 2008; Lee et al., 2010; Kim et al., 2011; Alers et al., 2012; Mack et al., 2012), but the relationship between the plant AMPK and autophagy is still unknown. In our study, *KIN10* overexpression activate autophagy pathway (**Figure 4**). Y2H assays suggested that KIN10 interacts with ATG1a and ATG13a *in vitro* (**Supplementary Figure S4**). However, we were unable to obtain further evidence of this interaction with BiFC and CoIP assays *in planta* (**Supplementary Figure S5**). We conclude that KIN10 positively regulates autophagy pathway through a possible unknown mechanism bypass ATG1/ATG13 protein complex. Alternatively, the activation of autophagy pathway by KIN10 overexpression may also be caused by inhibiting TOR activity, which is well-known to function as a negative regulator in autophagosome formation (Liu and Bassham, 2010). As suggested by Suttangkakul et al. (2011), the extent of ATG1a phosphorylation was highly regulated by the nutritional state through the action of upstream kinases and/or ATG1 autophosphorylation. Our results showed that, in response to starvation, overexpression of *KIN10* enhanced the phosphorylation of ATG1a (**Figure 5**) which supports the idea that autophagy may function downstream of the KIN10 kinase by directly or indirectly targeting ATG1 proteins for phosphorylation. In conclusion, our findings demonstrated that KIN10 is a positive regulator of autophagy in *Arabidopsis*.

## Author Contributions

SX and Q-FC designed the study. LC, Z-ZS, LH, F-NX, HQ, and L-JX carried out the experiments. SX, Q-FC, and LC analyzed the data. SX and LC wrote the manuscript.

## Conflict of Interest Statement

The authors declare that the research was conducted in the absence of any commercial or financial relationships that could be construed as a potential conflict of interest.

## Supplementary Material

FIGURE S1 Molecular identification of the *KIN10-OE* and *KIN10-RNAi* transgenic plants. **(A)** qRT-PCR analysis of *KIN10* transcript levels in 4-week-old wild type (WT), *OE-1*, *OE-2*, *RNAi-1*, and *RNAi-7* plants. Transcript levels relative to the wild type were normalized to the levels of *ACTIN2*. The data are means ± SD (*n* = 3) calculated from three biological replicates. ^∗∗^*P* < 0.01 by Student’s *t*-test. **(B)** Immunoblot analysis of KIN10 in 4-week-old WT, *OE-1*, *OE-2*, *RNAi-1*, and *RNAi-7* plants. Anti-KIN10 antibodies were used for immunoblotting. Coomassie blue-stained total proteins (Rubisco) are shown below the blot to indicate the amount of protein loaded per lane.

FIGURE S2 Overexpression of *KIN10* activates autophagy-related gene expression. Expression patterns of *ATGs* in the WT, *OE-1*, *OE-2*, *RNAi-1*, and *RNAi-7* plants. Total RNA was isolated from 4-week-old WT, *OE-1*, *OE-2*, *RNAi-1*, and *RNAi-7* plants under normal growth conditions. Transcript levels relative to the WT were normalized to that of *ACTIN2*. The data are means ± SD (*n* = 3) calculated from three biological replicates. ^∗∗^*P* < 0.01 by Student’s *t*-test.

FIGURE S3 *ATG1a* transcript levels in the *YFP-ATG1a* and *YFP-ATG1a/KIN10-OE* plants in response to carbon starvation. Total RNA was isolated from 7-day-old *YFP-ATG1a* and *YFP-ATG1a/KIN10-OE* transgenic plants grown on MS medium followed by carbon starvation for 0, 3, 6, 12, and 24 h. Transcript levels relative to *YFP-ATG1a* at 0 h were normalized to that of *ACTIN2*. The data are means ± SD (*n* = 3) calculated from three biological replicates. ^∗∗^*P* < 0.01 by Student’s *t*-test. Light gray bars indicate gene expression in the *YFP-ATG1a*, dark gray bars indicate gene expression in the *YFP-ATG1a/KIN10-OE*.

FIGURE S4 Yeast two-hybrid assays showing the physical interactions of KIN10 with autophagy-related proteins (ATGs). **(A)** Y2H assay of the interaction between KIN10 and ATG proteins (ATG1a, ATG1b, ATG1c, ATG6, ATG8e, ATG9, PI3K, ATG13a, and ATG13b). ATG1a, ATG1b, ATG1c, ATG6, ATG8e, ATG9, PI3K, ATG13a, and ATG13b bait constructs were fused to the DNA-binding domain (BD), and full-length KIN10 was fused to the activation domain (AD). Vectors containing the AD and BD were co-expressed in yeast strain YH109. Protein interactions were determined by a growth assay in a medium lacking Trp, Leu, His, and Ade, with 30 mM 3-amino-1,2,4-triazole which was added to repress self-activation. The vector containing the AD or BD alone served as the negative control. **(B)** Y2H assay of the interaction between the functional domains of KIN10 and ATG1a and ATG13a. ATG1a and ATG13a bait constructs were fused to the BD, and prey constructs were fused to the AD. The vector containing the BD alone served as the negative control. Full-length KIN10 contained a protein kinase domain (CD), a ubiquitin-associated domain (UBA) and a kinase associated domain 1 (KA1). Protein interaction was determined by a growth assay in a medium lacking Trp, Leu, His, and Ade (SD-Trp-Leu-His-Ade) supplemented with 30 mM 3-amino-1,2,4-triazole.

FIGURE S5 *In vivo* assays showing no interaction of KIN10 with ATG1a and ATG13a. **(A)** CoIP assay of the association between KIN10 and ATG1a/ATG13a. FLAG-tagged ATG1a/ATG13a and HA-tagged KIN10 (KIN10-HA) was transiently expressed in protoplasts from wild-type *Arabidopsis* and immunoprecipitated by FLAG affinity agarose beads. **(B)** BiFC assay of KIN10 interaction with ATG1a and ATG13a in *Arabidopsis* protoplast cells. **(C)** BiFC assay of the interaction between functional domains of KIN10 and ATG1a/ATG13a in *Arabidopsis* protoplast cells. The split nYFP and cYFP fused to KIN10 and ATG1a/ATG13 were coexpressed in leaf protoplasts. nYFP/cYFP and ATG6-nYFP/TRAF1a-cYFP vectors were similarly co-expressed as negative and positive controls. Confocal images obtained from YFP, auto-fluorescent chlorophyll, and bright-field are shown. Bars = 20 μm.
