# Supplementary figures and images for: The AMP-Activated Protein Kinase KIN10 Is Involved in the Regulation of Autophagy in Arabidopsis
Source: Front Plant Sci. 2017 Jul 10;8:1201. doi: 10.3389/fpls.2017.01201 (PMC5502289; doi:10.3389/fpls.2017.01201)

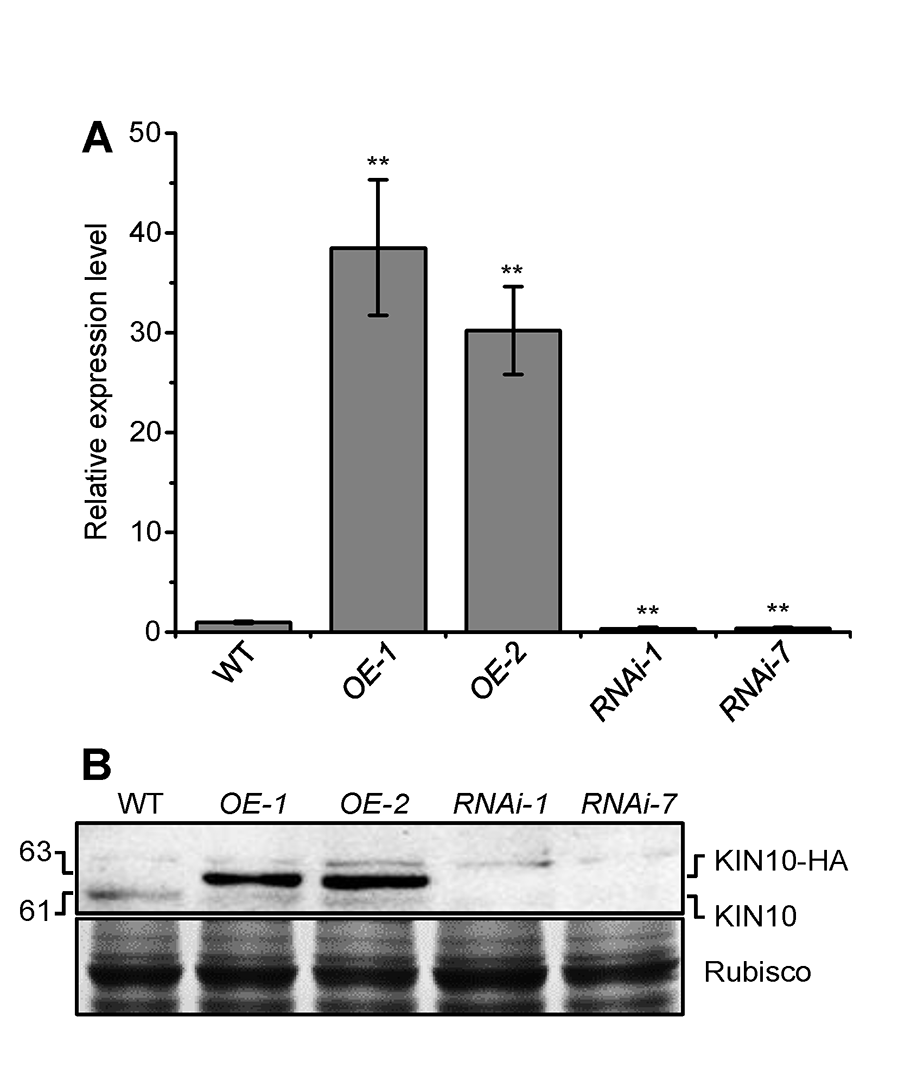

Supplement: FIGURE S1 — Molecular identification of the KIN10-OE and KIN10-RNAi transgenic plants. (A) qRT-PCR analysis of KIN10 transcript levels in 4-week-old wild type (WT), OE-1, OE-2, RNAi-1, and RNAi-7 plants. Transcript levels relative to the wild type were normalized to the levels of ACTIN2. The data are means ± SD (n = 3) calculated from three biological replicates. ∗∗P < 0.01 by Student’s t-test. (B) Immunoblot analysis of KIN10 in 4-week-old WT, OE-1, OE-2, RNAi-1, and RNAi-7 plants. Anti-KIN10 antibodies were used for immunoblotting. Coomassie blue-stained total proteins (Rubisco) are shown below the blot to indicate the amount of protein loaded per lane. [file Image_1.TIF]

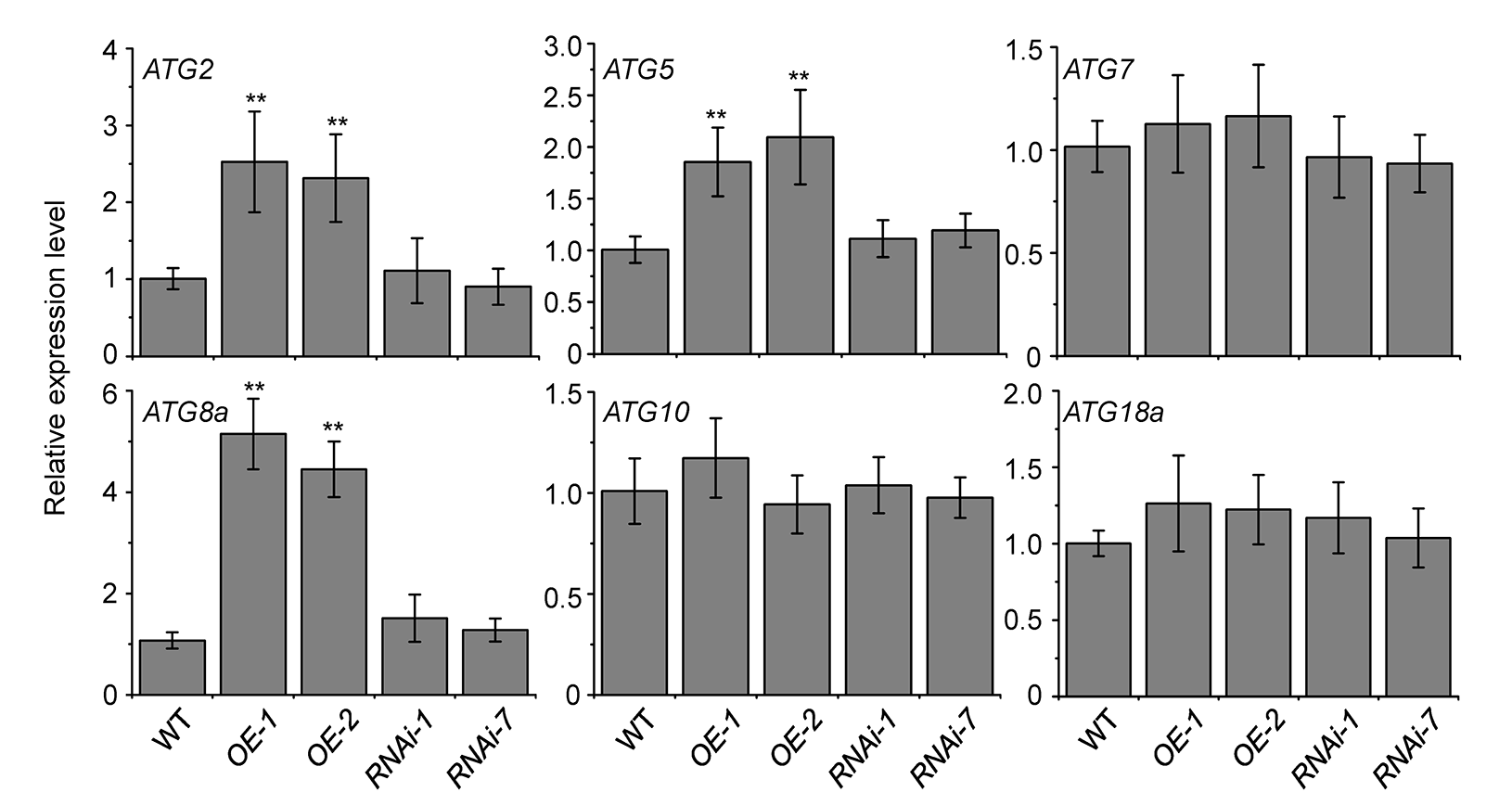

Supplement: FIGURE S2 — Overexpression of KIN10 activates autophagy-related gene expression. Expression patterns of ATGs in the WT, OE-1, OE-2, RNAi-1, and RNAi-7 plants. Total RNA was isolated from 4-week-old WT, OE-1, OE-2, RNAi-1, and RNAi-7 plants under normal growth conditions. Transcript levels relative to the WT were normalized to that of ACTIN2. The data are means ± SD (n = 3) calculated from three biological replicates. ∗∗P < 0.01 by Student’s t-test. [file Image_2.TIF]

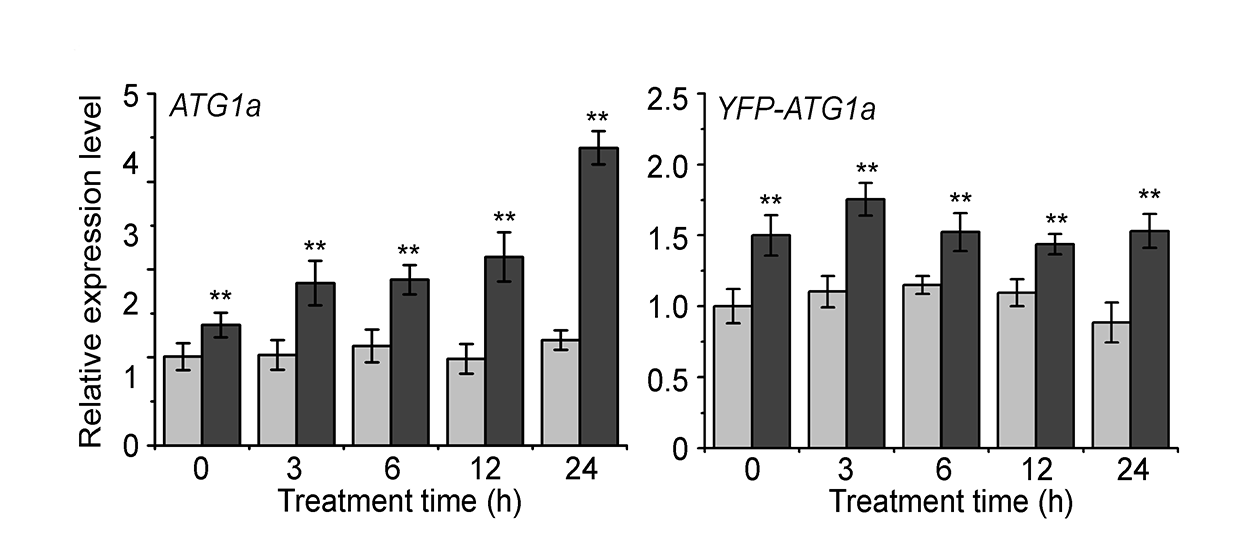

Supplement: FIGURE S3 — ATG1a transcript levels in the YFP-ATG1a and YFP-ATG1a/KIN10-OE plants in response to carbon starvation. Total RNA was isolated from 7-day-old YFP-ATG1a and YFP-ATG1a/KIN10-OE transgenic plants grown on MS medium followed by carbon starvation for 0, 3, 6, 12, and 24 h. Transcript levels relative to YFP-ATG1a at 0 h were normalized to that of ACTIN2. The data are means ± SD (n = 3) calculated from three biological replicates. ∗∗P < 0.01 by Student’s t-test. Light gray bars indicate gene expression in the YFP-ATG1a, dark gray bars indicate gene expression in the YFP-ATG1a/KIN10-OE. [file Image_3.TIF]

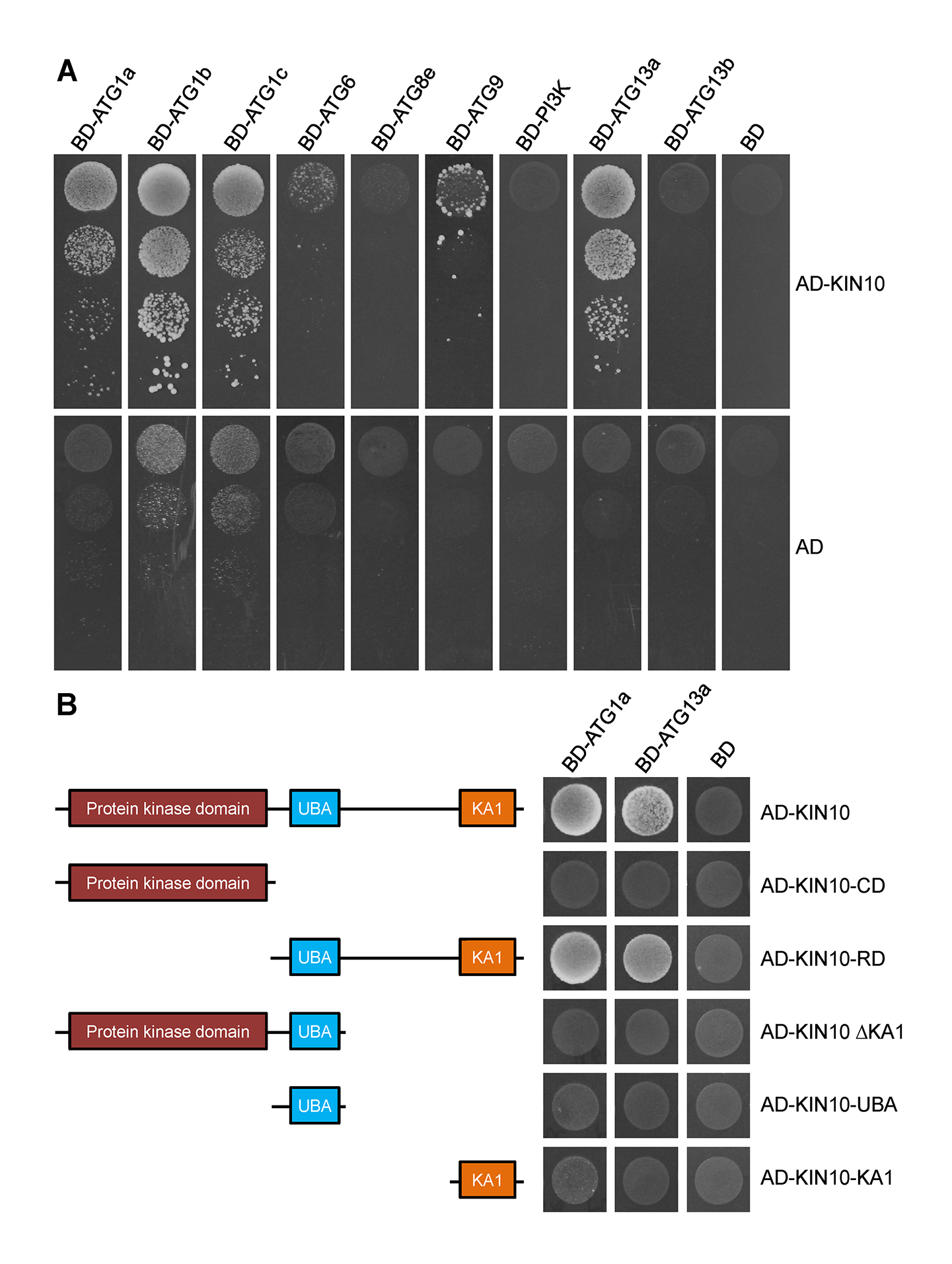

Supplement: FIGURE S4 — Yeast two-hybrid assays showing the physical interactions of KIN10 with autophagy-related proteins (ATGs). (A) Y2H assay of the interaction between KIN10 and ATG proteins (ATG1a, ATG1b, ATG1c, ATG6, ATG8e, ATG9, PI3K, ATG13a, and ATG13b). ATG1a, ATG1b, ATG1c, ATG6, ATG8e, ATG9, PI3K, ATG13a, and ATG13b bait constructs were fused to the DNA-binding domain (BD), and full-length KIN10 was fused to the activation domain (AD). Vectors containing the AD and BD were co-expressed in yeast strain YH109. Protein interactions were determined by a growth assay in a medium lacking Trp, Leu, His, and Ade, with 30 mM 3-amino-1,2,4-triazole which was added to repress self-activation. The vector containing the AD or BD alone served as the negative control. (B) Y2H assay of the interaction between the functional domains of KIN10 and ATG1a and ATG13a. ATG1a and ATG13a bait constructs were fused to the BD, and prey constructs were fused to the AD. The vector containing the BD alone served as the negative control. Full-length KIN10 contained a protein kinase domain (CD), a ubiquitin-associated domain (UBA) and a kinase associated domain 1 (KA1). Protein interaction was determined by a growth assay in a medium lacking Trp, Leu, His, and Ade (SD-Trp-Leu-His-Ade) supplemented with 30 mM 3-amino-1,2,4-triazole. [file Image_4.TIF]

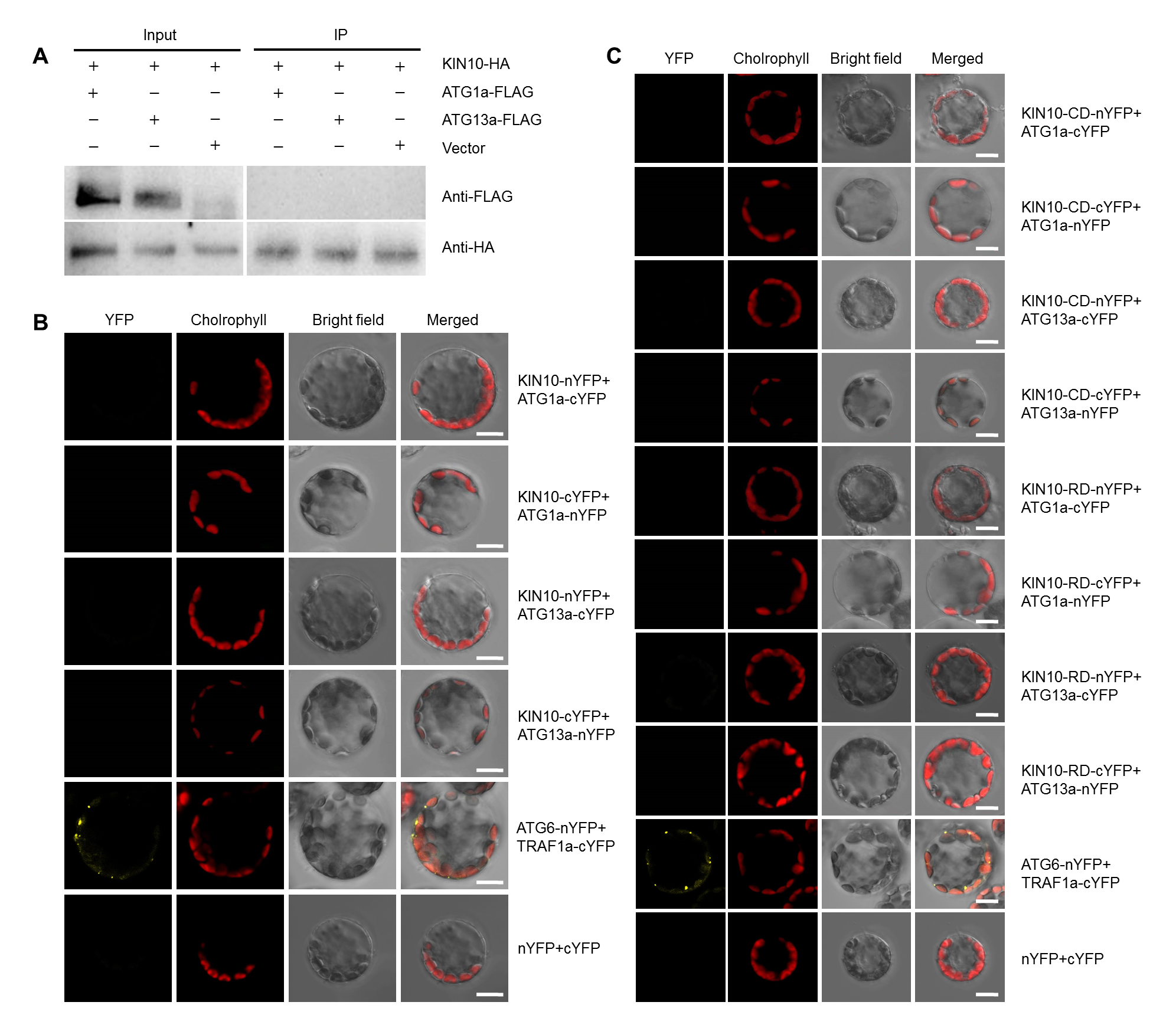

Supplement: FIGURE S5 — In vivo assays showing no interaction of KIN10 with ATG1a and ATG13a. (A) CoIP assay of the association between KIN10 and ATG1a/ATG13a. FLAG-tagged ATG1a/ATG13a and HA-tagged KIN10 (KIN10-HA) was transiently expressed in protoplasts from wild-type Arabidopsis and immunoprecipitated by FLAG affinity agarose beads. (B) BiFC assay of KIN10 interaction with ATG1a and ATG13a in Arabidopsis protoplast cells. (C) BiFC assay of the interaction between functional domains of KIN10 and ATG1a/ATG13a in Arabidopsis protoplast cells. The split nYFP and cYFP fused to KIN10 and ATG1a/ATG13 were coexpressed in leaf protoplasts. nYFP/cYFP and ATG6-nYFP/TRAF1a-cYFP vectors were similarly co-expressed as negative and positive controls. Confocal images obtained from YFP, auto-fluorescent chlorophyll, and bright-field are shown. Bars = 20 μm. [file Image_5.TIF]
