# Supplementary material for: The AMP-Activated Protein Kinase KIN10 Is Involved in the Regulation of Autophagy in Arabidopsis
Source: Front Plant Sci. 2017 Jul 10;8:1201. doi: 10.3389/fpls.2017.01201 (PMC5502289; doi:10.3389/fpls.2017.01201)
Supplement: Supplementary file 6 [file Table_1.PDF]

Supplemental Table 1. Sequence of Primers Used in This Study.

Gene	Name	Sequence (5'→3')	Usage
ACTIN2	ML1124	CCCGCTATGTATGTCGC	qRT-PCR
	ML1125	AAGGTCAAGACGGAGGAT	
KIN10	XS057	GTGAAGATAGCTGAGCATGC	qRT-PCR
	XS058	TGAGGTCTCTGTGAACCACC	
ATG2	XS1029	AATGGATAGCAAGTGGAAGC	qRT-PCR
	XS1030	AGATAGACCTACCGTTAGCC	
ATG5	XS1031	ACTGATACCATGTGAAGGAG	qRT-PCR
	XS1032	GTATAGGCATCAAGATCACC	
ATG7	XS1033	GAAGATTGTCTAGGTCGTGG	qRT-PCR
	XS1034	CCTGCTTTCTCTTGTATCGG	
ATG8a	XS033	GCAGAGACTAATCGAATCGC	qRT-PCR
	XS034	CAAGCAACGGTAAGAGATCC	
ATG10	XS1035	ATCATACAAGGTTCCTGTGC	qRT-PCR
	XS1036	GATGTAGCTTGAACCATGGC	

ATG18a	XS224	AGATCATGCTTGCTTCGCTG	qRT-PCR
	XS225	AGAGTTCTCCGATACATCGG	
ATG1a	XS529	GGAAATCACTGTTGCCCTCC	qRT-PCR
	XS530	TATGAAACGCTTGCTGGTCG	
YFP-ATG1a	XS3870	GCACGACTTCTTCAAGTCCG	qRT-PCR
	XS3871	CCTCGATGTTGTGGCGGATC	
KIN10	XS617	GGAATTCATGTTCAAACGAGTAGATG	AD-KIN10 constraction
	XS618	CGGGATCCTCAGAGGACTCGGAG	
ATG1a	XS611	GGAATTCATGGAGTCGGCACGAC	BD-ATG1a constraction
	XS612	CGGGATCCCTAAAGATGAGACCGACG	
ATG1b	XS613	GGAATTCATGGCTCAGTCGTCGTTG	BD-ATG1b constraction
	XS614	CGGGATCCTCATTGCTTCTGAGG	
ATG1c	XS615	GGAATTCATGGCTCAGTTCACG	BD-ATG1c constraction
	XS616	CGGGATCCTTATGATTGTCTGTTCATC	
ATG6	XS2183	CATGGAGGCCGAATTCGATTACAAGGATGATGATGATAAG	BD-ATG6 constraction
	XS2184	GGATCCCCGGGAATTCCTAAGTTTTTTTACATGAAGGCT	

ATG8e	XS2757	GGCCATGGAGGCCGAATTCATGAATAAAGGAAGCATCTTTAAGATG	BD-ATG8e constraction
	XS2758	GCTGCAGGTCGACGGATCCTTAGATTGAAGAAGCACCGAATG	
ATG9	XS2930	CATGGAGGCCGAATTCATGATGAGCAGTGGGCATAAGG	BD-ATG9 constraction
	XS2931	GCAGGTCGACGGATCCTCACCGTAATGTGGTGCTTGATGT	
PI3K	XS2932	CATGGAGGCCGAATTCATGGGTGCGAACGAGTTTCG	BD-PI3K constraction
	XS2933	GCAGGTCGACGGATCCTCAACGCCAGTATTGAGCCCAT	
ATG13a	XS623	GGAATTCATGGATTTTCCAGAG	BD-ATG13a constraction
	XS624	CGGGATCCTCAGTGGACGCGAGTTG	
ATG13b	XS625	TCCCCCGGGGATGTCATCTTCACAC	BD-ATG13b constraction
	XS626	CGGGATCCTCAAACATCAAAGGGTG	
KIN10	XS3244	GAGGCCAGTGAATTCATGTTCAAACGAGTAGATG	AD-KIN10-CD constraction
	XS3245	ACCCGGGTGGAATTCTCACCTCGGAAGATGAGCTTGG	
KIN10	XS3246	GAGGCCAGTGAATTCCCGAGGTATTTAGCTGTTCC	AD-KIN10-RD constraction
	XS3247	ACCCGGGTGGAATTCTCAGAGGACTCGGAGCTG	
KIN10	XS3244	GAGGCCAGTGAATTCATGTTCAAACGAGTAGATG	AD-KIN10-ΔKA1 constraction
	XS3344	ACCCGGGTGGAATTCTCAATAACCACTAGAGGCACG	

KIN10	XS3345	GAGGCCAGTGAATTCTATTTAGCTGTTCCTCCTCC	AD-KIN10-UBA constraction
	XS3346	ACCCGGGTGGAATTCTCAATAACCACTAGAGGCACG	
KIN10	XS3347	GAGGCCAGTGAATTCATAGAGAACGAAGCAGCTG	AD-KIN10-KA1 constraction
	XS3247	ACCCGGGTGGAATTCTCAGAGGACTCGGAGCTG	
KIN10	XS2031	TGATTAACAGGGATCCATGTTCAAACGAGTAGATGAG	KIN10-HA constraction
	XS2032	GTCGTATGGGTAAGGCCTGAGGACTCGGAGCTGAGC	
ATG1a	XS2027	TCTGATTAACAGGGATCCATGGAGTCGGCACGAC	ATG1a-FLAG constraction
	XS2028	TCCTTGTAGTCAGAAGGCCTAAGATGAGACCGACGATG	
ATG13a	XS2029	TCTGATTAACAGGGATCCATGGATTTTCCAGAGAATTTGC	ATG13a-FLAG constraction
	XS2030	TCCTTGTAGTCAGAAGGCCTGTGGACGCGAGTTGG	
ATG1a	XS4014	CTTGCTCCGTGGATCCATGGAGTCGGCACG	ATG1a-cYFP/nYFP constraction
	XS4015	TACTATCGATGGATCCAAGATGAGACCGACGATG	
ATG13a	XS4020	CTTGCTCCGTGGATCCATGGATTTTCCAGAGAATTTGC	ATG13a-cYFP/nYFP constraction
	XS4021	TACTATCGATGGATCCGTGGACGCGAGTTGG	
KIN10	XS4235	CTTGCTCCGTGGATCCATGTTCAAACGAGTAGATGAGTT	KIN10-cYFP/nYFP constraction
	XS4238	TACTATCGATGGATCCGAGGACTCGGAGCTGAG	

KIN10	XS4235	CTTGCTCCGTGGATCCATGTTCAAACGAGTAGATGAGTT	KIN10-CD-cYFP/nYFP constraction
	XS4236	TACTATCGATGGATCCCCTCGGAAGATGAGCTTGG	
KIN10	XS4237	CTTGCTCCGTGGATCCATGCCGAGGTATTTAGCTGTTCC	KIN10-RD-cYFP/nYFP constraction
	XS4238	TACTATCGATGGATCCGAGGACTCGGAGCTGAG	
	LB1	GCCTTTTCAGAAATGGATAAATAGCCTTGCTT	T-DNA primer
